# Significant Alterations in Phospholipid Content and Composition Contributing to Metabolic Dysfunction in ADMSC-Derived Adipocytes

**DOI:** 10.3390/cells15191725

**Published:** 2026-09-22

**Authors:** Bartłomiej Łukaszuk, Elżbieta Supruniuk, Jan Górski, Monika Naumowicz, Marcin Zając, Adrian Chabowski, Agnieszka Mikłosz

**Affiliations:** 1 Department of Physiology, Medical University of Bialystok, Mickiewicza 2C, 15-222 Białystok, Poland; elzbieta.supruniuk@umb.edu.pl (E.S.); adrian.chabowski@umb.edu.pl (A.C.); agnieszka.miklosz@umb.edu.pl (A.M.); 2 Faculty of Health Sciences, University of Lomza, 18-400 Lomza, Poland; 3 Department of Physical Chemistry, Faculty of Chemistry, University of Bialystok, Ciolkowskiego 1K, 15-245 Białystok, Poland; monikan@uwb.edu.pl; 4 Doctoral School of Exact and Natural Sciences, University of Bialystok, Ciolkowskiego 1K, 15-245 Białystok, Poland

**Keywords:** stem cells, adipocytes, cellular research, phospholipids, memory effect

## Abstract

Adipose-derived mesenchymal stem cells (ADMSCs) are a crucial source of new adipocytes and play a significant role in regulating adipose tissue physiology. Interestingly, mature adipocytes differentiated from the ADMSCs maintain some characteristic features of their respective depots as well as the metabolic status of the donor patients. The above is true with respect to the cells’ secretory profile and their glycerolipid and sphingolipid composition. In the current paper, we focused our attention on the phospholipid profile of the adipocytes derived from ADMSCs, with a particular emphasis placed on how obesity and metabolic syndrome affect their composition. Surprisingly, the cells displayed a significantly greater (even up to 10-fold) overall phospholipid concentration when compared with the primary human adipocytes described in the literature. The phenomenon may be explained by a geometrical property known as surface-to-volume ratio, which dictates that the total surface area of many small cells (our adipocytes) is greater than the total area of less numerous but more voluminous cells in vivo. Interestingly, also a donor’s metabolic status can influence the characteristics of these fat cells. The study found that subcutaneous adipocytes, when derived from patients with obesity and metabolic syndrome, had an increased concentration of phosphatidylcholine (PC), phosphatidylethanolamine (PE), phosphatidylinositol (PI), and sphingomyelin (SM). The above-mentioned changes often reflect the ones found in vivo and may well be the early metabolic disarrangements that occur in the progress of obesity.

## 1. Introduction

Obesity is a complex medical condition characterized by an excessive accumulation of body fat that increases the risk of developing serious health issues like hypertension, dyslipidemia, metabolic syndrome, or type 2 diabetes mellitus (T2DM) [1]. Obesity develops from a prolonged imbalance between the energy intake from food and its expenditure through physical activity. Its surplus is stockpiled as fat in the form of energy-rich triacylglycerols (TAG) [2]. White adipose tissue (WAT) is distributed throughout the body with its two main depots found in subcutaneous (SAT) and visceral (VAT) regions. These depots have distinct characteristics and roles in metabolic regulation [3]. In normal physiological conditions, SAT composes majority of the tissue mass and functions as a thermal insulator, preventing excessive heat loss [4]. Importantly, in the case of need (temporarily increased lipid supply), the tissue is capable of expanding, thus performing the action of a ‘metabolic sink’. However, in obesity, more than two-thirds of the circulating non-esterified fatty acids (NEFAs) originate from the upper body subcutaneous fat depots, which implies that a chronic energy surplus may lead to a ‘lipid spillover’ into peripheral tissues (like skeletal muscle), a condition known as ectopic lipid accumulation, and its deleterious consequences (i.e., peripheral insulin resistance) [4,5,6]. Interestingly, even though in healthy, lean individuals VAT is usually less voluminous, it is still considered to be the more metabolically active of the two depots [7]. For example, studies suggest that VAT tends to release more cytokines to its surroundings (endocrine and paracrine function), as demonstrated by us [8] and other teams [9]. Khan and co-authors compared conditioned media from omental (visceral) and subcutaneous fat of obese patients and found that the omental fat produced significantly higher levels of certain inflammatory molecules, like interleukins (IL-6, IL-8, IL-12p70, and IL-13), tumor necrosis factor alpha (TNF-α), and other adipokines (MMP-1, TBX2, and PGE2) [9]. The above is of vital importance once we consider that this type of fat (VAT) is localized in the vicinity of the internal organs and its secretome is constantly released into the portal circulation, thus affecting the liver and, therefore, whole-body glucose metabolism [7].

In recent years, there has been a shift toward exploring fibroblast-like adipose-derived mesenchymal stem cells (ADMSCs) [10]. ADMSCs are a class of progenitor cells with the potential to differentiate into multiple lineages (like osteocytes, chondrocytes, or adipocytes) [10]. Because of that they are considered as a potentially limitless source of new fat cells. This facility is believed to be crucial for the tissue’s ability to adapt and perform its biological functions. Our recent research demonstrated that the adipocytes differentiated from the ADMSCs display an interesting lipid [11,12] and adipokine profile [8] that parallels with the one found in the tissue in vivo [9,13]. First of all, we observed that the cells maintain the characteristic features of their respective depots. In line with that notion, the mature adipocytes differentiated from ADMSCs of visceral ancestry (visADMSCs) were larger and showed less granularity in the cytoplasm than their counterparts of subcutaneous origin (subADMSCs) [11]. Moreover, also the lipid profile reflected the one found in humans [13], as the cells in the visceral tended to have less triacyl- and diacylglycerols (DAG), but more ceramides (CER) [11,12]. Additionally, the secretory profile of the differentiated adipocytes mirrors the tissue they originate from, with the cells derived from visADMSCs exhibiting a greater release of adipokines [9]. Interestingly, the adipocytes also appear to preserve, at least to an extent, the metabolic status of the donor patients, as we observed an increased fatty acid transporters content (total and/or cell surface CD36/SR-B2 and FATP4 expression) together with their greater intracellular influx (3H-palmitic acid uptake) in the cells derived from obese subjects. The above translated into greater TAG and DAG concentrations that were visible in the cells of subcutaneous progeny but only when they were obtained from obese patients with metabolic syndrome. Of note, this over-accumulation is often considered to be a hallmark of the condition [14] (Figure 1).

The previously discovered phenomena [8,11,12] prompted us to deepen our understanding of the topic, with respect to the phospholipids profile of the adipocytes differentiated from ADMSCs [11]. Phospholipids are a group of amphipathic molecules that form the biological membranes [15]. Phospholipids are believed to be involved in many physiological processes, like cellular signaling, inflammation, or aging [16,17,18]. Their presence in the adipose tissue is also of importance, as previous studies suggest their implication in the development of the tissue malfunctioning. For instance, Zeghari et al. [19] demonstrated the existence of a relationship between the cell membrane phospholipids and hyperinsulinemia. The authors found strong positive correlations between the adipose plasma membrane sphingomyelin (SM) content and fasting plasma insulin level (SM vs. FPI, r = 0.698, *p* <0.005), as well as the HOMA index (SM vs. HOMA-IR, r = 0.637, *p* < 0.01), that were present in obese women (BMI > 30 kg/m^2^). Of note, phosphatidylcholine (PC) and phosphatidylethanolamine (PE) were also positively associated with the investigated parameters (PC vs. FPI: r = 0.531 and *p* < 0.05; PE vs. HOMA-IR: r = 0.514 and *p* < 0.05). The authors attributed their findings to a simultaneous drop in the expression of the peroxisome proliferator-activated receptor-γ (SM vs. PPARγ: r = −0.553, *p* < 0.05), a known regulator of the insulin signaling cascade and glucose transporter (GLUT-4) translocation [20]. Moreover, it appears that not only the total amount of phospholipids, but also their composition, can influence various cellular processes and biological outcomes. For example, the available literature data show that an increased fatty acid chain length increases plasma membrane thickness, whereas their saturation status affects its fluidity (↑unsaturated fatty acids → ↑fluidity), thus changing its mechanical and biological properties [21].

Given the above, here we focused on examining phospholipid metabolism in the mature adipocytes that have been differentiated from ADMSCs. For this purpose, we used a previously successfully developed model [11], in which we analyzed selected phospholipids, i.e., phosphatidylcholine (PC), phosphatidylethanolamine (PE), phosphatidylserine (PS), phosphatidylinositol (PI), and sphingomyelin (SM). The analysis encompassed mature adipocytes of subcutaneous and visceral origin obtained from the ADMSCs of patients with different metabolic status: lean, and obese with and without metabolic syndrome.

## 2. Materials and Methods

### 2.1. The Participants and Adipose-Derived Mesenchymal Stem Cells (ADMSCs) Collection

Ahead of any experiments, the study protocol was approved by the Ethics Committee of the Medical University of Bialystok (permission R-I-002/187/2017). All the participants gave their permission to participate in the research by signing written informed consent forms. All the women were under the care of the First Department of General and Endocrine Surgery at the University Hospital in Bialystok. The tissue collection was conducted as described before [11]. Twelve postmenopausal women who qualified for the study were equally divided into three groups (*n* = 4 per group): (1) lean (BMI < 25 kg/m^2^) control (group designation: Lean), (2) obese (BMI > 40 kg/m^2^), but without the concomitant metabolic syndrome (group designation: Obese(−)), and (3) obese (BMI > 40 kg/m^2^) with the concomitant metabolic syndrome (group designation: Obese(+)). White adipose tissue samples (~2–4 g) were obtained during the scheduled procedures (laparoscopic cholecystectomy in the lean group and sleeve gastrectomy in the obese groups). The tissues were immediately put into PBS (phosphate-buffered saline; PAN-Biotech, Aidenbach, Germany) and moved to a proper facility for further proper conduct. The ADMSCs were isolated according to the previously proved protocol [11]. Briefly, the tissues were washed in PBS; afterwards, they underwent the removal of visible blood vessels. Next, the samples were chopped and minced for easier digestion in a collagenase solution (250 U/mL, NB 4G Proved Grade, Serva, Heidelberg, Germany). Naturally occurring primary adipocytes were removed from a sample by using a cell strainer (pluriSelect Life Sciene, Leipzig, Germany) with a 500 µm mesh size. The stromal vascular fraction that did pass through the strainer was centrifuged. The pellet was mixed with an erythrocyte lysis buffer (Thermo Fisher Scientific, Waltham, MA, USA). The pellet underwent a series of purification steps. First, it was filtered twice using cell strainers with mesh sizes of 200 µm and 20 µm. Following the filtration, the pellet was subjected to centrifugation (600× *g*) for 5 min. The obtained pellet was rich in adipose-derived mesenchymal stem cells that were suspended in the mesenchymal stem cell medium (MSCM; ScienCell Research Laboratories, Carlsbad, CA, USA). The medium was supplemented with 5% *v*/*v* fetal bovine serum (FBS; Thermo Fisher Scientific, Waltham, MA, USA) and 1% *v*/*v* antibiotic/antimycotic (PAN-Biotech, Aidenbach, Germany). The medium was regularly replaced (3–4 times per week) with a fresh one until the cultivated cells reached the proper density (80–90% of confluency). Flow cytometry was applied to confirm the obtained cells’ viability and ADMSCs’ identity. Moreover, the cells’ trilineage (adipo-, osteo-, and chondro-genic) differentiation potential was also confirmed, as described in our previous research [11,22].

### 2.2. Differentiation of ADMSCs into Mature Adipocytes

A detailed description of all the applied procedures can be found elsewhere [11]. Briefly, the obtained ADMSCs were seeded on the appropriate cell culture plates using the aforementioned MSCM. Only when the confluency approached 90% was the adipogenesis triggered. To that end, the medium was replaced by MADM (mesenchymal stem cell adipogenic differentiation medium; ScienCell Research Laboratories, Carlsbad, CA, USA) with the addition of FBS (5% *v*/*v*) and antibiotic/antimycotic solution (1% *v*/*v*). The medium was changed to a fresh one roughly 2 times per week. The differentiation process took approximately 2–3 weeks; afterwards, the obtained mature adipocytes could be subjected to subsequent analyses. Importantly, the proper course of differentiation was corroborated by continuous microscopic observation of the cells and Oil Red-O (ORO) staining (Merck, Darmstadt, Germany), as described in our previous research [11,22].

### 2.3. Determination of Phospholipid Content in the Mature Adipocytes Differentiated from ADMSCs

The intracellular content of the selected phospholipid species, i.e., phosphatidylcholine (PC), phosphatidylethanolamine (PE), phosphatidylserine (PS), phosphatidylinositol (PI), and sphingomyelin (SM), was assessed by the means of gas–liquid chromatography (GLC). Briefly, the samples underwent lipid extraction by a well-established protocol originally described by Folch and co-authors [23]. For that process, we used a chloroform–methanol mixture (2:1 by volume) containing butylated hydroxytoluene as an antioxidant and heptadecanoic acid as an internal standard. The phospholipids were obtained after an overnight extraction and were separated into particular fractions of interest by thin-layer chromatography (TLC). The separation took place on glass chromatographic plates that had been covered with silica gel (Silica Plate 60, 0.25 mm; Merck, Darmstadt, Germany). A chloroform/methanol/acetic acid solution (50:37.5:3.5, *v*/*v*/*v*) was used as a resolvent. Each plate contained all the analyzed groups and the reference standard. Lipid bands were made visible by spraying plates with a 0.2% solution of 2′7′-dichlorofluorescin in methanol and viewed under ultraviolet light using respective standards on the plates. Once the process was complete, plate bands corresponding to the selected phospholipid fractions were scraped off with a margin of error (an exemplary TLC plate can be found in Supplementary Figure S1). The obtained powder was subjected to elution and subsequent transmethylation using a solution of boron trifluoride in methanol (14% *v*/*v*). In the next step, the obtained samples were extracted using pentane solution and evaporated under a nitrogen stream. Finally, hexane was added to the samples. The identification and quantification of the individual fatty acid methyl esters was performed based on the standard retention times using a Hewlett-Packard 5890 Series II gas chromatograph (Agilent Technologies, Santa Clara, CA, USA) with a HP-INNOWax capillary column (Agilent Technologies, Santa Clara, CA, USA). The amount of total phospholipids (PC, PE, PS, PI, and SM) in the adipocytes was estimated as the sum of the individual fatty acid species found in a sample and expressed in nmol per mg of protein.

### 2.4. Electrophoretic Light Scattering Measurements

The zeta potential (ζ) of the adipocytes was assessed using a Zetasizer Nano ZS analyzer (Malvern Instruments Ltd., Malvern, UK) based on the electrophoretic light scattering (ELS) method, as described in detail elsewhere [24]. Briefly, disposable folded capillary zeta cells (Malvern DTS 1070) were used to perform the experiment. All measurements were carried out with respect to the acidity/basicity of the surrounding environment (as expressed by pH) using a WTW InoLab pH 720 laboratory meter (WTW, Weinheim, Germany). For that, the samples were initially suspended in a sodium chloride solution (Cm = 0.155 mmol/L) and titrated to the desired pH value (range [2.5–10]) with sodium hydroxide or hydrochloric acid. The measurements of the potential were performed every 0.3 pH units. Six electrophoretic mobility measurements (each covering 100–200 series with a duration of 2 s), for every sample, at a given pH value were performed.

The zeta potential (ζ) of the adipocytes was calculated as established in the literature [25,26], i.e., from the electrophoretic mobility (Henry’s equation) according to the following formula:

𝜁 = [3·𝜂·𝜇]/[2·𝜀·𝜀0·𝑓(𝜅𝑎)],

where:

μ—electrophoretic mobility,

η—viscosity of the aqueous solution,

ε—relative permittivity of the medium,

*ε*0—the permittivity of free space,

ƒ(*κa*)—Henry’s function.

### 2.5. Statistical Analysis

Prior to statistical analysis, data preprocessing was performed using LibreOffice Calc (ver. 24.2). Proper statistical evaluation was conducted with the R software (ver. 4.3.3). The obtained numerical data were analyzed using three-way ANOVA, with the three factors of interest being tissue, obesity, and the presence of metabolic syndrome. Subsequent between-group comparisons were performed with either the Student’s *t*-test or Wilcoxon rank sum test. The choice of the method was based on the fulfillment of normality (Shapiro–Wilk test) and homogeneity of variances (Fligner–Killeen test) criteria. The obtained *p*-values were corrected with the Benjamini–Hochberg procedure, and a *p*-value lower than 0.05 was deemed significant.

## 3. Results

The distribution of phospholipids within a given group is depicted in Figure 2. Overall, the most abundant phospholipid species were phosphatidylcholine and phosphatidylethanolamine. The remaining three classes of phospholipids (phosphatidylinositol, phosphatidylserine, and sphingomyelin) were clearly less numerous. Their (PI, PS, and PC) concentrations were roughly similar between the groups (Figure 2).

**Figure 2:**
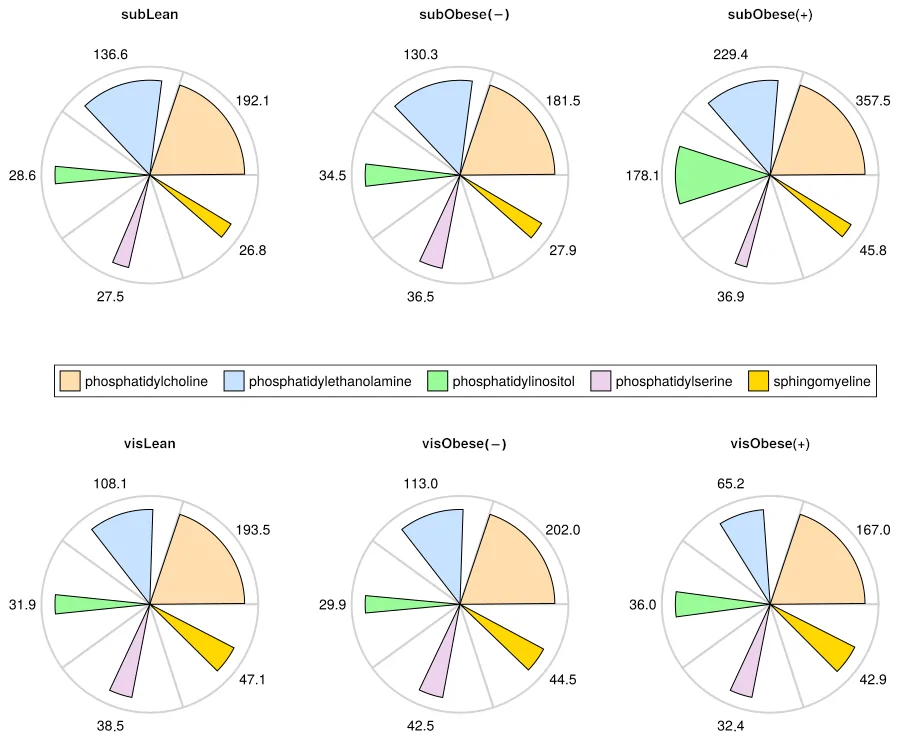
Overall distribution of phospholipid species in the mature adipocytes differentiated from ADMSCs of female patients with different metabolic status. Data are depicted as pie charts with a slice area proportional to the mean concentration of a given phospholipid within a given group (number next to a slice and bar height in Figure 3, Figure 4, Figure 5, Figure 6 and Figure 7). Designation of the groups: Obese(−)—obese without metabolic syndrome patients; Obese(+)—obese with metabolic syndrome patients; vis—visceral adipose tissue; sub—subcutaneous adipose tissue.

**Figure 3:**
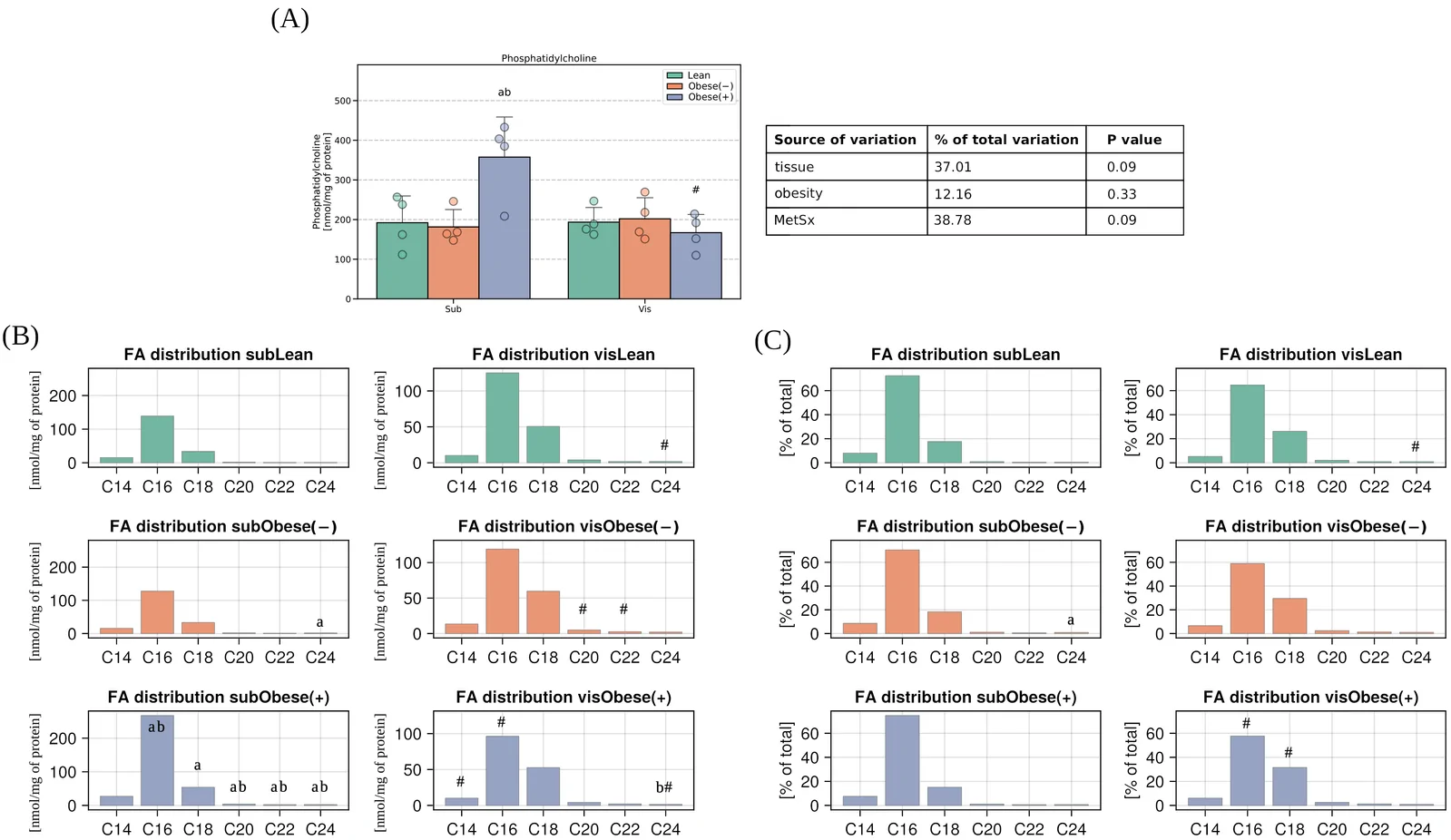
Phosphatidylcholine (PC) concentration in the mature adipocytes differentiated from ADMSCs of subcutaneous and visceral provenance. (**A**) Total concentration of PC. (**B**) Histogram of distribution of fatty acid chain lengths (C14–C24) that build PC in nmol/mg of protein. (**C**) Histogram of distribution of fatty acid chain lengths (C14–C24) that build PC as a percentage of the total. Data are depicted as mean (bar height) and standard deviation (whiskers), *n* (per group) = 4. a—Difference vs. lean (*p* < 0.05) within the tissue type; b—difference vs. Obese(−) (*p* < 0.05) within the tissue type; #—difference vis vs. sub (*p* < 0.05), but within the patient metabolic type. Obese(−): adipocytes differentiated from ADMSCs of patients with obesity but without metabolic syndrome; Obese(+): adipocytes differentiated from ADMSCs of patients with obesity with accompanying metabolic syndrome.

**Figure 4:**
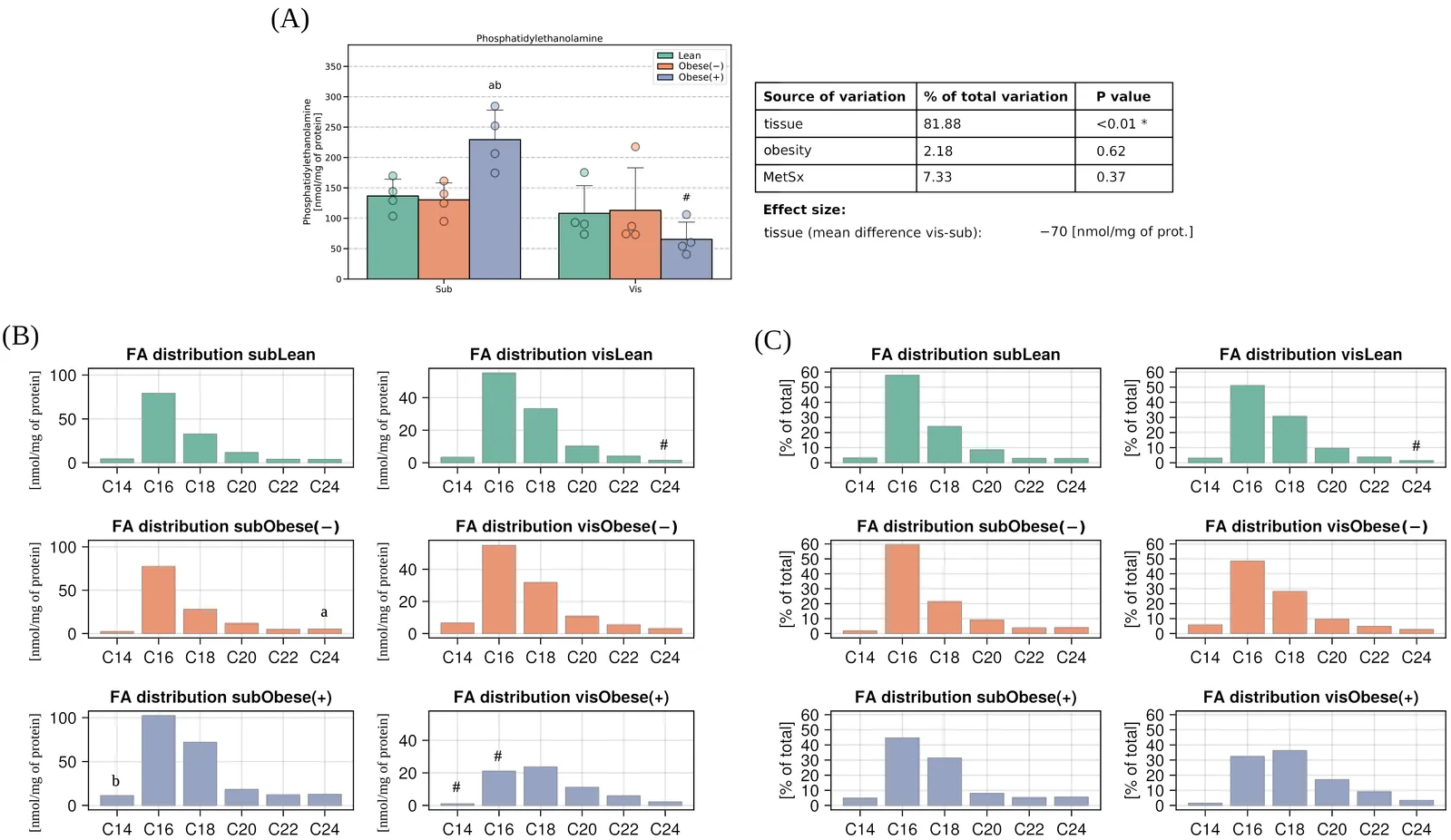
Phosphatidylethanolamine (PE) concentration in the mature adipocytes differentiated from ADMSCs of subcutaneous and visceral provenance. (**A**) Total concentration of PE. (**B**) Histogram of distribution of fatty acid chain lengths (C14–C24) that build PE in nmol/mg of protein. (**C**) Histogram of distribution of fatty acid chain lengths (C14–C24) that build PE as a percentage of the total. Data are depicted as mean (bar height) and standard deviation (whiskers), *n* (per group) = 4. a—difference vs. lean (*p* < 0.05) within the tissue type; b—difference vs. Obese(−) (*p* < 0.05) within the tissue type; #—difference vis vs. sub (*p* < 0.05), but within the patient metabolic type. Obese(−): adipocytes differentiated from ADMSCs of patients with obesity but without metabolic syndrome; Obese(+): adipocytes differentiated from ADMSCs of patients with obesity with accompanying metabolic syndrome. *—Statistical significance (*p* < 0.05) of the examined factor in three-way ANOVA.

**Figure 5:**
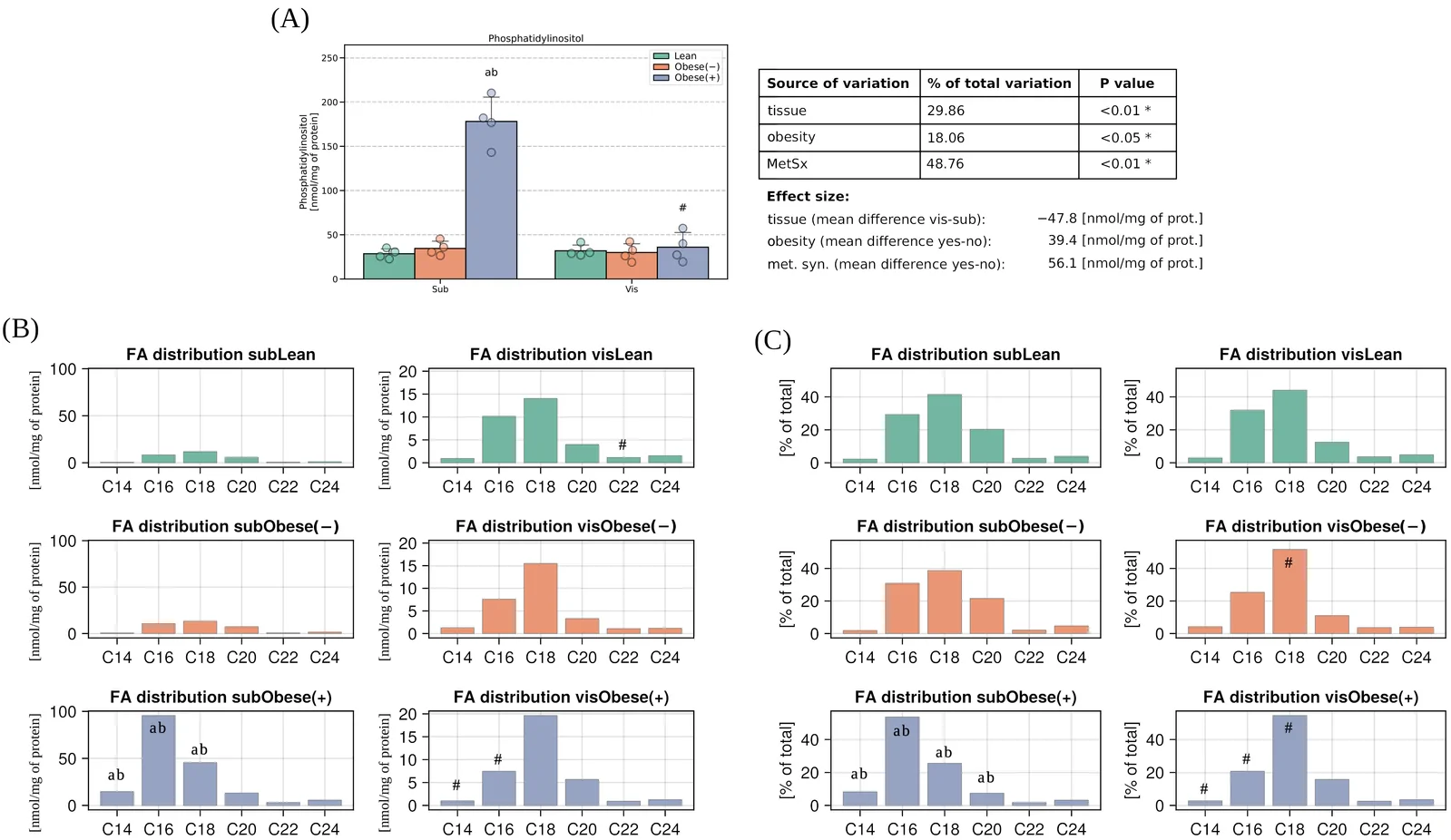
Phosphatidylinositol (PI) concentration in the mature adipocytes differentiated from ADMSCs of subcutaneous and visceral provenance. (**A**) Total concentration of PI. (**B**) Histogram of distribution of fatty acid chain lengths (C14–C24) that build PI in nmol/mg of protein. (**C**) Histogram of distribution of fatty acid chain lengths (C14–C24) that build PI as a percentage of the total. Data are depicted as mean (bar height) and standard deviation (whiskers), *n* (per group) = 4. a—Difference vs. lean (*p* < 0.05) within the tissue type; b—difference vs. Obese(−) (*p* < 0.05) within the tissue type; #—difference vis vs. sub (*p* < 0.05), but within the patient metabolic type. Obese(−): adipocytes differentiated from ADMSCs of patients with obesity but without metabolic syndrome; Obese(+): adipocytes differentiated from ADMSCs of patients with obesity with accompanying metabolic syndrome. *—Statistical significance (*p* < 0.05) of the examined factor in three-way ANOVA.

**Figure 6:**
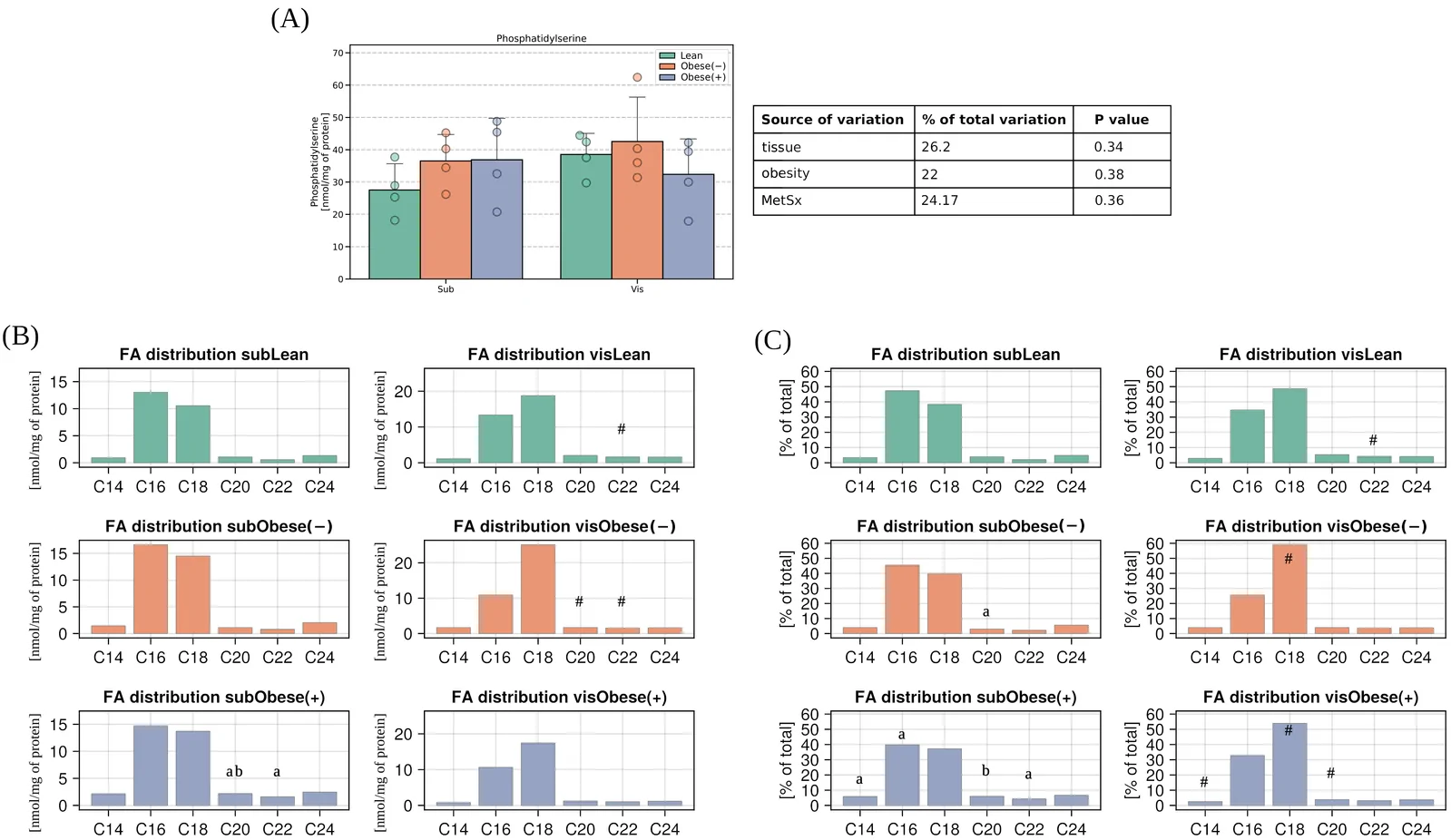
Phosphatidylserine (PS) concentration in the mature adipocytes differentiated from ADMSCs of subcutaneous and visceral provenance. (**A**) Total concentration of PS. (**B**) Histogram of distribution of fatty acid chain lengths (C14–C24) that build PS in nmol/mg of protein. (**C**) Histogram of distribution of fatty acid chain lengths (C14–C24) that build PS as a percentage of the total. Data are depicted as mean (bar height) and standard deviation (whiskers), *n* (per group) = 4. a—difference vs. lean (*p* < 0.05) within the tissue type; b—difference vs. Obese(−) (*p* < 0.05) within the tissue type; #—difference vis vs. sub (*p* < 0.05), but within the patient metabolic type. Obese(−): adipocytes differentiated from ADMSCs of patients with obesity but without metabolic syndrome; Obese(+): adipocytes differentiated from ADMSCs of patients with obesity with accompanying metabolic syndrome.

**Figure 7:**
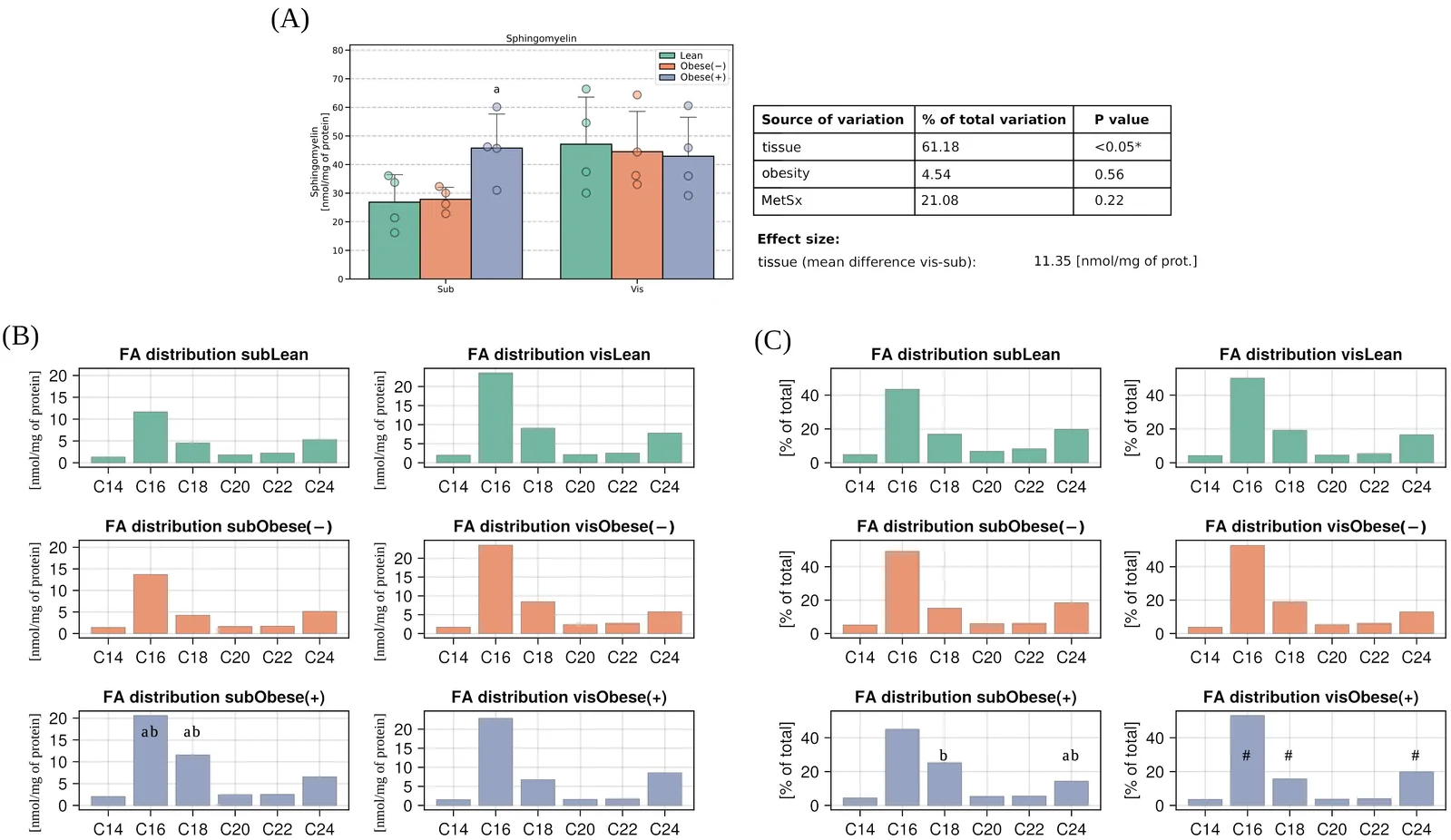
Sphingomyelin (SM) concentration in the mature adipocytes differentiated from ADMSCs of subcutaneous and visceral provenance. (**A**) Total concentration of SM. (**B**) Histogram of distribution of fatty acid chain lengths (C14–C24) that build SM in nmol/mg of protein. (**C**) Histogram of distribution of fatty acid chain lengths (C14–C24) that build SM as a percentage of the total. Data are depicted as mean (bar height) and standard deviation (whiskers), *n* (per group) = 4. a—Difference vs. lean (*p* < 0.05) within the tissue type; b—difference vs. Obese(−) (*p* < 0.05) within the tissue type; #—difference vis vs. sub (*p* < 0.05), but within the patient metabolic type. Obese(−): adipocytes differentiated from ADMSCs of patients with obesity but without metabolic syndrome; Obese(+): adipocytes differentiated from ADMSCs of patients with obesity with accompanying metabolic syndrome. *—Statistical significance (*p* < 0.05) of the examined factor in three-way ANOVA.

### 3.1. Phosphatidylcholine

In general, the content of phosphatidylcholine was relatively stable between the examined groups, as none of the studied factors had systematic influence on its total concentration in the cells (*p* > 0.05; Figure 3A). The only statistically significant difference was the subObese(+) group that displayed a higher level of the phospholipid when compared with subLean (+86%, *p* < 0.05) and subObese(−) groups (+97%, *p* < 0.05; Figure 3A). The above was caused by a shift toward greater accumulation of fatty acids of 16 to 24 carbons per chain (Figure 3B), with the greatest accretion (in absolute numbers) observed for palmitic (C16:0) and palmitooleic (C16:1, n7) species. Despite that notion, the percentage distribution of fatty acid chain lengths was relatively stable among the investigated groups (Figure 3C). Particular fatty acid compositions of phosphatidylcholine can be found in the Supplementary Data.

### 3.2. Phosphatidylethanolamine

Visceral adipocytes have significantly lower levels of phosphatidylethanolamine (PE) compared to subcutaneous adipocytes, with an average difference of ~70 nmol/mg protein (*p* < 0.05; Figure 4A). Moreover, between-group comparisons revealed a greater phosphatidylethanolamine concentration in the subObese(+) group when compared to subLean (+68%, *p* < 0.05) and subObese(−) groups (+76%, *p* < 0.05; Figure 4A). The above was mostly caused by an increased concentration of palmitooleic (C16:1), stearic (C18:0), and linoleic (C18:2) fatty acids that individually, due to relatively high data variability, failed to reach the level of statistical significance (*p* > 0.05; Figure 4B). An opposite trend was observed in the cells of visceral origin, as visObese(+) had a lower concentration of phosphatidylethanolamine compared to visLean (−40%, *p* > 0.05) and visObese(−) (−42%, *p* > 0.05; Figure 4A). Nevertheless, the relative distribution of fatty acids with respect to their chain lengths was relatively stable (Figure 4C), with a trend (*p* > 0.05) toward smaller percentages of C16 species observed in subObese(+) and visObese(+) groups (Figure 4C). Particular fatty acid compositions of phosphatidylethanolamine can be found in the Supplementary Data.

### 3.3. Phosphatidylinositol

As for the total content of phosphatidylinositol, it was relatively stable, except for the clear peak (~178 nmol/mg of protein) in the subObese(+) group that stood out from the rest of the observations (*p* < 0.05) that were in the range ~28–36 nmol/mg of protein. The aforementioned spike likely inflated the effect sizes for the analyzed factors in the three-way ANOVA (Figure 5A). It was caused by greater accumulation of fatty acids, especially those of 14 to 18 carbons in the chain (Figure 5B), i.e., myristic (C14:0), palmitic (C16:0), palmitooleic (C16:1), and oleic (C18:1) acids. Besides the reported distinct fatty acid profile of subObese(+) cells, other groups displayed a roughly similar composition of the analyzed fatty acids (Figure 5B,C). Particular fatty acid compositions of phosphatidylinositol can be found in the Supplementary Data.

### 3.4. Phosphatidylserine

Regarding cellular phosphatidylserine content, we did not detect systematic nor between-group differences (Figure 6A). The above was also reflected in the distribution of fatty acids constituting phosphatidylserine, as only a few minor changes were observed (Figure 6B). For instance, we noticed an increased concentration (+100–160%, *p* < 0.05) of C20 and C22 fatty acids in the subObese(+) adipocytes when compared with their counterparts from subLean and subObese(−) groups (Figure 6B). As regards the percentage distribution of fatty acid chains, the subObese(+) group appeared to have a lower content of C16 fatty acids and a higher amount of C14, C20, and C22 fatty acids (Figure 6C). On the other hand, a between-tissue comparison indicates that in the adipocytes of visceral origin, phosphatidylserine is composed primarily of eighteen-carbon (C18) fatty acid chains, rather than sixteen-carbon (C16) chains, as in their subcutaneous counterparts (Figure 6C). Particular fatty acid compositions of phosphatidylserine can be found in the Supplementary Data.

### 3.5. Sphingomyelin

Detailed analysis demonstrated a systematic effect of tissue type (~61% of data variability, *p* < 0.05) on the total sphingomyelin level (Figure 7A). On average, the adipocytes of visceral origin had an ~11 nmol/mg of protein greater level of the lipid. Moreover, we observed a clear peak in SM concentration in the cells derived from subADMSCs of the patients with metabolic syndrome (+71% for subObese(+) vs. subLean, *p* < 0.05, and +64% for subObese(+) vs. subObese(−), *p* > 0.05; Figure 7A). The above was caused by the accumulation of 16- and 18-carbon fatty acids (*p* < 0.05; Figure 7B). With respect to the percentage distribution of fatty acid chain lengths, it was relatively stable, with only minor non-directional changes visible (Figure 7C). Particular fatty acid compositions of sphingomyelin can be found in the Supplementary Data.

### 3.6. Zeta Potential

The assessment of the zeta potential (ζ-potential) was done using electrophoretic light scatter and the results are presented in Figure 8. The curves depicting the relationship between the electrokinetic potential and pH of the solution had a similar shape for all the examined groups (subLean, subObese(−), and subObese(+); Figure 8A). The isoelectric point for the observations is located within pH 4.2–4.35. The electrokinetic potential drops from 10 mV to −18 mV with the increase in the concentration of OH- ions in the solution. Similarly, the curves for the cells of visceral provenance (Figure 8B) also did not differ much between the analyzed groups. The zeta potential in the VAT groups was within a narrower range of 9 mV to −12 mV, and the isoelectric point was located at pH equal to 4.3–4.45. Overall, we did not detect any clear trends in the analyzed data.

A one-figure graphical summary of the most important results is depicted in Figure 9.

## 4. Discussion

In the current study we sought to thoroughly examine the overall phospholipid profile of the mature adipocytes differentiated from ADMSCs. Since, to our knowledge, there are no studies investigating the phospholipid profile in mature adipocytes differentiated from ADMSCs, we compared our data with that of human adipose tissue. This comparison helps to understand the characteristics of these cells and how they relate to naturally occurring adipose tissue. Careful scrutiny of the recently published papers revealed some phenotypic differences between our cells and those found in vivo. Palavcini et al. [27], for instance, examined the adipocytes derived from the subcutaneous adipose tissue of seven obese individuals with type 2 diabetes mellitus. The authors assessed their glycerophospholipid profile and a modulatory effect exerted on thereof by pioglitazone (an insulin-sensitizing drug). Of interest, the researchers assessed the concentrations of sphingomyelin (SM), phosphatidylethanolamine (PE), phosphatidylcholine (PC), phosphatidylserine (PS), and phosphatidylinositol (PI) [27]. The reported cellular content was in the range of ~4 to ~30 nmol/mg of protein, with PE and PC being at the upper end, whereas PI was at the lower end of the spectrum. The above stands in contrast to our research, as the amount of phospholipids in the subObese(+) group, which is metabolically similar to the examined patients mentioned above, ranged from ~40 to ~350 nmol/mg of protein. Clearly, the values obtained in the current investigation are from a few to even 10-fold greater than those from the study of Palavcini and co-authors [27]. One possible explanation for such a discrepancy may be the significantly lower protein content commonly observed in cell lines compared to respective tissue homogenates. Such a difference was previously reported, i.e., in the case of hepatocytes [28] or skeletal muscles [29]. Similar reports can be found also for the adipose tissue. Yu and Scherrer [30] used the bicinchoninic acid (BCA) protein assay to evaluate protein concentration in mouse subcutaneous white adipose tissue (sWAT). The above was, on average, equal to 4.13 µg/µL. On the other hand, Sailer and co-authors [31] placed its (protein) concentration in 3T3-L1 cells (adipocytes of murine origin) to be ~1 mg/mL (equivalent of ~1 µg/µL). This aligns with our observations, as we noticed roughly 2–3 times greater protein concentration in the human subcutaneous adipose tissue lysate (not displayed). This could partially account for the reported differences, as the phospholipid content is expressed in nmol/mg of protein so the smaller denominator (less protein found in the adipocytes differentiated form ADMSCs) will lead to a higher overall concentration of the lipids observed in our cell culture. This is because the same amount of phospholipid (nmol) is being divided by a smaller protein mass (mg), which results in a larger quotient. Still, despite that fact, it appears that our adipocytes do have a higher total phospholipid concentration than their counterparts found in vivo. At first glance, this seems counterintuitive, as this class of lipids is found primarily in biological membranes, and their surface area should positively correlate with cell size. Moreover, more voluminous adipocytes are expected to be found in the living human body. In line with that notion, Honecker et al. estimated that the average area of an adipocyte in vivo is within the range of 20,000–30,000 µm^2^ [32]. The value is significantly greater than the one found in the mature adipocytes differentiated from ADMSCs, as reported in our previous study [11], which was in the range of hundreds µm^2^. The above is only an apparent issue, as we must remember that in reality the cells are three-dimensional objects, and the whole problem can be reduced to a geometric property called the surface-to-volume ratio that also has applications in a medical setting [33]. Briefly, calculations based on mathematical formulas for different objects indicate that smaller objects have a greater surface-to-volume ratio [33], which in our case would mean a greater plasma membrane surface area per a unit of encapsulated volume. Nevertheless, in the context of the current study, this indicates that the total area of the plasma membranes of many small cells (the adipocytes derived from ADMSCs in this study) should be greater than the total surface area of a few voluminous cells (the adipocytes found in vivo). The logical consequence of the above is a greater expected concentration of phospholipids (building blocks of the plasma membrane), as found in the current study. The above considerations are also supported by another observation. The cells in living organisms (in vivo) accumulate triacylglycerols within a large, single, membrane-bound lipid droplet [34]. On the other hand, as reported in our previous research [11], the adipocytes differentiated from ADMSCs are characterized by many small lipid droplets. Therefore, based on the previously discussed mathematical properties, also in a cell culture, the total surface area of the intracellular plasma membranes is expected to be greater, which further supports our observations, despite the previously mentioned difference with the human tissue in vivo [27]. Surprisingly, this could partially account for another finding in this study. Previously, based on the microscopic analysis and flow cytometry, we have shown that visceral adipocytes, on average, tend to be larger and have fewer granularities (likely lipid droplets) in their cytoplasm compared to the subcutaneous adipocytes [11]. This could explain the systematic effect of the tissue type on the phospholipid content reported here, i.e., smaller concentration of the molecules in the cells differentiated from visADMSCs.

Further analysis of our data revealed a few clear patterns and some more debatable smaller ones. We observed a quite consistent arrangement in the total phospholipid concentrations among the different examined lipid classes. The above is uniformly visible in the peaks present in the figures for phosphatidylcholine (PC), phosphatidylethanolamine (PE), phosphatidylinositol (PI), and sphingomyelin (SM). Interestingly, it is solely present in the cells obtained from obese patients with metabolic syndrome (MetSx), but only when the adipocytes were derived from the ADMSCs of subcutaneous origin. This could be a potential early hallmark of the condition. However, one would expect to observe such pronounced change in visceral fat cells, as many studies report omental fat tissue as a useful marker of cardiometabolic diseases and metabolic syndrome [35,36]. Still, metabolic disturbances seem to affect the subcutaneous tissue first, as indicated by Arner and Rydén [37]. The authors observed that individuals with a higher body fat percentage tend to have reduced lipid turnover in their adipocytes, meaning they store lipids at a slower rate and experience greater accumulation of lipids within those cells. This slower turnover and increased lipid accumulation are key factors in the development and progression of obesity. Interestingly, in the subcutaneous depot, the dysregulation occurs already in the overweight individuals, while the visceral location appears to be affected only in the case of the patients with severe obesity [37]. The above is corroborated by the study of Zacharia et al., where paired SAT and VAT biopsies demonstrated greater accumulation of tri- and di-acylglycerols in the former [13]. This observation was also reflected in our previous study [11], in which a pronounced amount of the mentioned lipids was observed in the cells from the subObese(+) group.

As regards the phospholipids (PC, PE, PI, and SM) for which an increase in their total content was observed in the cells derived from the obese patients with metabolic syndrome, literature data indicate that the molecules may be implicated in the development of said condition [38,39,40,41]. For instance, Wentworth et al. [38] examined the adipose tissue of 29 non-diabetic (fasting glucose level < 7 mmol/L) morbidly obese (BMI > 40 kg/m^2^) women, 13 of whom were hyperinsulinemic. Of note, hyperinsulinemia and glucose intolerance are considered to be hallmarks of metabolic syndrome. The omental tissue of the group with hyperinsulinemia was characterized by the presence of hypertrophied adipocytes infiltrated by macrophages. Importantly, the fat cells also had an increased content of GM3 ganglioside and phosphatidylethanolamine (PE), which the authors considered to be markers of insulin resistance [38]. Moreover, the researchers observed an association between the elevated levels of PE in the blood and reduced insulin sensitivity in the individuals [38]. Similarly, sphingomyelin also appears to be directly implicated in the tissue’s glucose homeostasis. Previous research by Long and Pekala indicates that in 3T3-L1 adipocytes, SM is an important source of ceramide, a bioactive lipid that inhibits the expression and stability of GLUT-4 (insulin-dependent glucose-sensitive transporter) mRNA, potentially further reducing GLUT-4 protein levels [41]. In line with that notion, we noticed an increased level of ceramide in the mature adipocytes from the subObese(+) group in our previous study [12]. This could be an early sign of disturbed insulin signaling in the cells. Moreover, phosphatidylcholine also seems to be implicated in cellular insulin signaling. He and co-authors investigated the actions of LPCAT3, an enzyme involved in PC synthesis, on the adipose tissue phenotype. They found that its higher activity in the fat tissue of ob/ob, db/db, or even high-fat-diet-fed mice (classical models of obesity) resulted in cell membrane remodeling and an impaired insulin signaling pathway [40]. However, the picture that stems from the research is not as clear cut and unequivocal as it may appear, as some studies have associated the pathological phenomena (obesity and insulin resistance) with changes in the saturation status of the fatty acid species composing the phospholipids [27,40]. The aforementioned paper by He et al. [40] indicates the addition of polyunsaturated PCs into the human or mouse adipocytes in vitro as a key factor responsible for the disruption of the insulin signaling pathway. In line with that notion, research by Palavicini on pioglitazone demonstrated that the adipocytes treated with that insulin sensitizer had a significantly decreased PUFA fraction, but an increased amount of saturated fatty acids and shortened chain lengths in phospholipids [27]. Nevertheless, in our study we were unable to reliably detect any correlation between the fatty acid chain length or the saturation status in any of the examined phospholipid classes. One possible explanation for this phenomenon is that obesity and a high-fat-diet regime translate into an elevated blood plasma lipid composition [42] that significantly influences the adipose tissue phenotype [43,44]. However, the above did not occur in our cells, as the adipocytes were cultivated in the same standard medium that contained adequate amounts of the necessary nutrients, including free fatty acids.

## 5. Conclusions

In summary, we expect this to be the first study that thoroughly examines phospholipid metabolism in the mature adipocytes differentiated from ADMSCs of women with different metabolic status (lean, and obese with or without metabolic syndrome). Our investigation allowed us to make a few interesting observations. Firstly, we found significantly increased (even up to 10-fold) overall phospholipid levels when we compared our cells with the ones found in human adipose tissue. We believe the phenomenon is best explained by a geometrical property known as surface-to-volume ratio, which dictates that the total surface area of many small cells (our adipocytes) is greater than the total area of less numerous but more voluminous cells in vivo. Moreover, we observed that a person’s metabolic condition can have a downstream effect on the characteristics and functions of the cells derived from different tissues. We noticed that the adipocytes of subcutaneous provenance that stemmed from the patients with obesity and metabolic syndrome had an increased concentration of phosphatidylcholine, phosphatidylethanolamine, phosphatidylinositol, and sphingomyelin. The abovementioned changes may well be the early metabolic disarrangements that occur in the progress of obesity and predispose the cells toward reduced insulin sensitivity.

## 6. Limitations

The current paper is a preliminary study; thus, it has a few shortcomings that should be addressed in future research. Firstly, the number of biological samples, although prevalent in the field of stem cell research, is considered modest in absolute quantities. A follow-up study of greater size should allow to counterbalance the between-patient data variability visible on some graphs and provide additional insights. Secondly, all the cells were cultivated in medium with FBS that alone contains a bunch of phospholipids for which no “background” control was run. Thirdly, the nutritional status (e.g., omnivorous vs. vegan) of the donor patients was not recorded, and this could potentially influence the lipid composition of the cells investigated in this study.

## Figures and Tables

**Figure 1:**
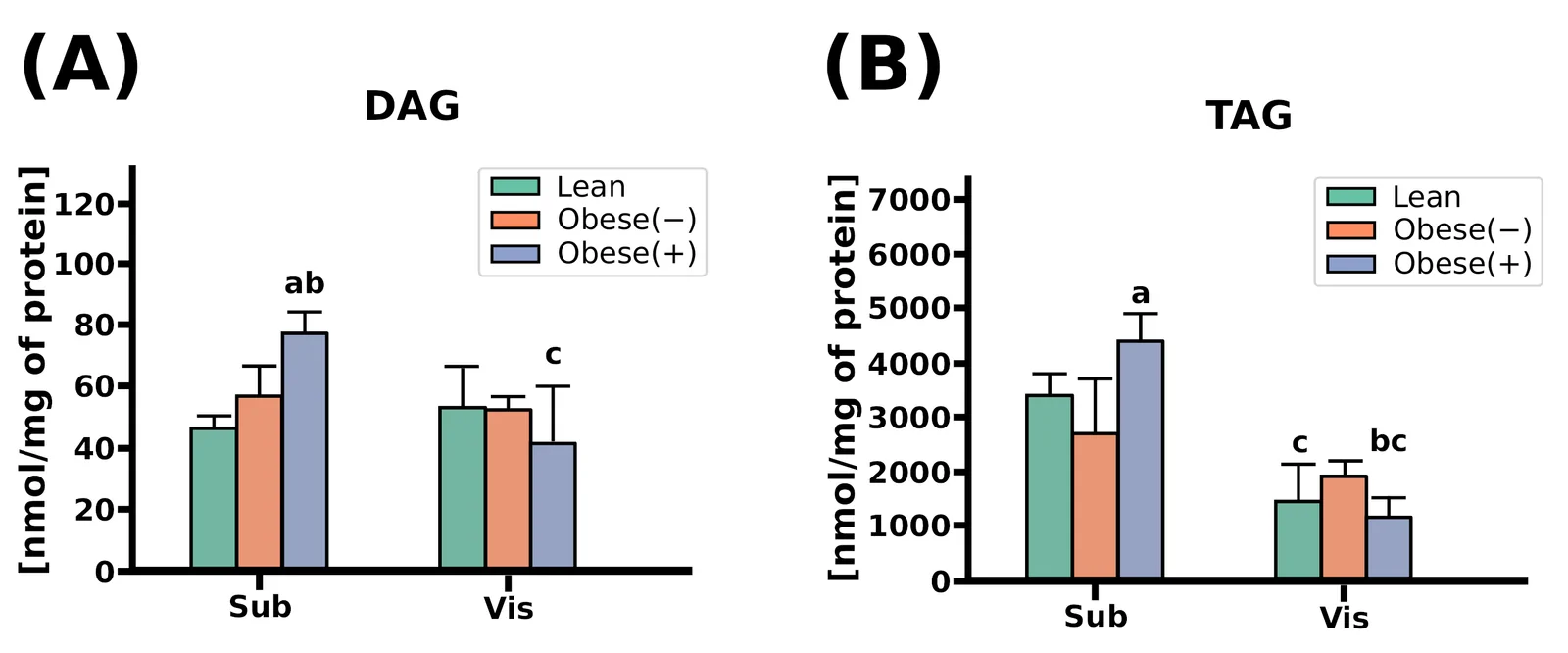
Summary of glycerolipids content in the mature adipocytes differentiated from ADMSCs of female patients with different metabolic status (based on the data contained in PMID: 35563741). (**A**) Diacylglycerol (DAG) concentration and (**B**) triacylglycerol (TAG) concentration. Data are depicted as mean (bar height) and standard deviation (whiskers). a—Difference vs. Lean group in the studied tissue; b—difference vs. Obese(−) group in the studied tissue; c—difference between adipocytes differentiated from visADMSCs vs. subADMSCs within the patient metabolic status. Designation of the groups: Obese(−)—obese without metabolic syndrome patients; Obese(+)—obese with metabolic syndrome patients; vis—visceral adipose tissue; sub—subcutaneous adipose tissue.

**Figure 8:**
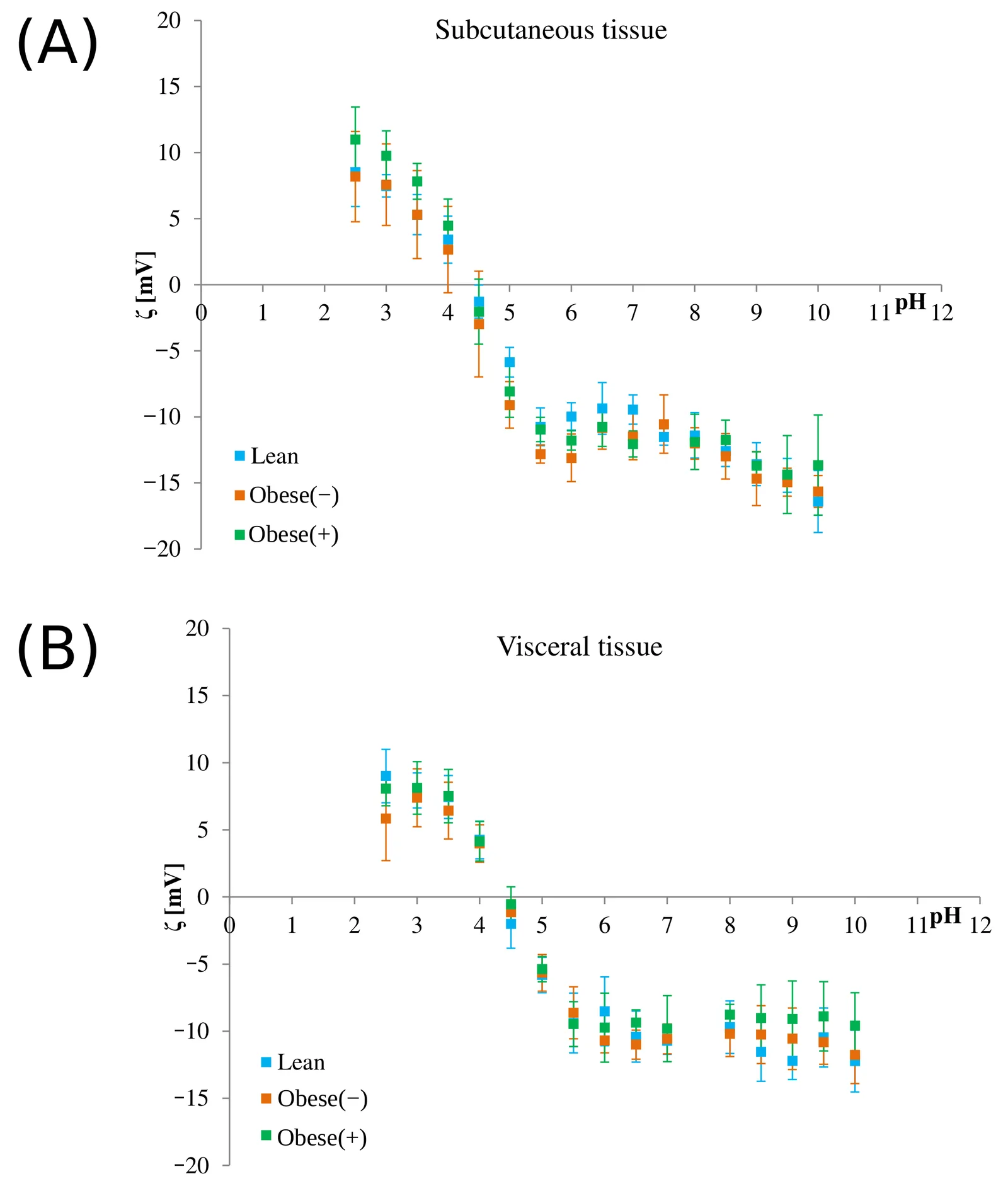
The relationship between the zeta potential and pH in the mature adipocytes differentiated from ADMSCs of subcutaneous (**A**) and visceral (**B**) provenance. Obese(−): adipocytes differentiated from ADMSCs of patients with obesity but without metabolic syndrome; Obese(+): adipocytes differentiated from ADMSCs of patients with obesity with accompanying metabolic syndrome.

**Figure 9:**
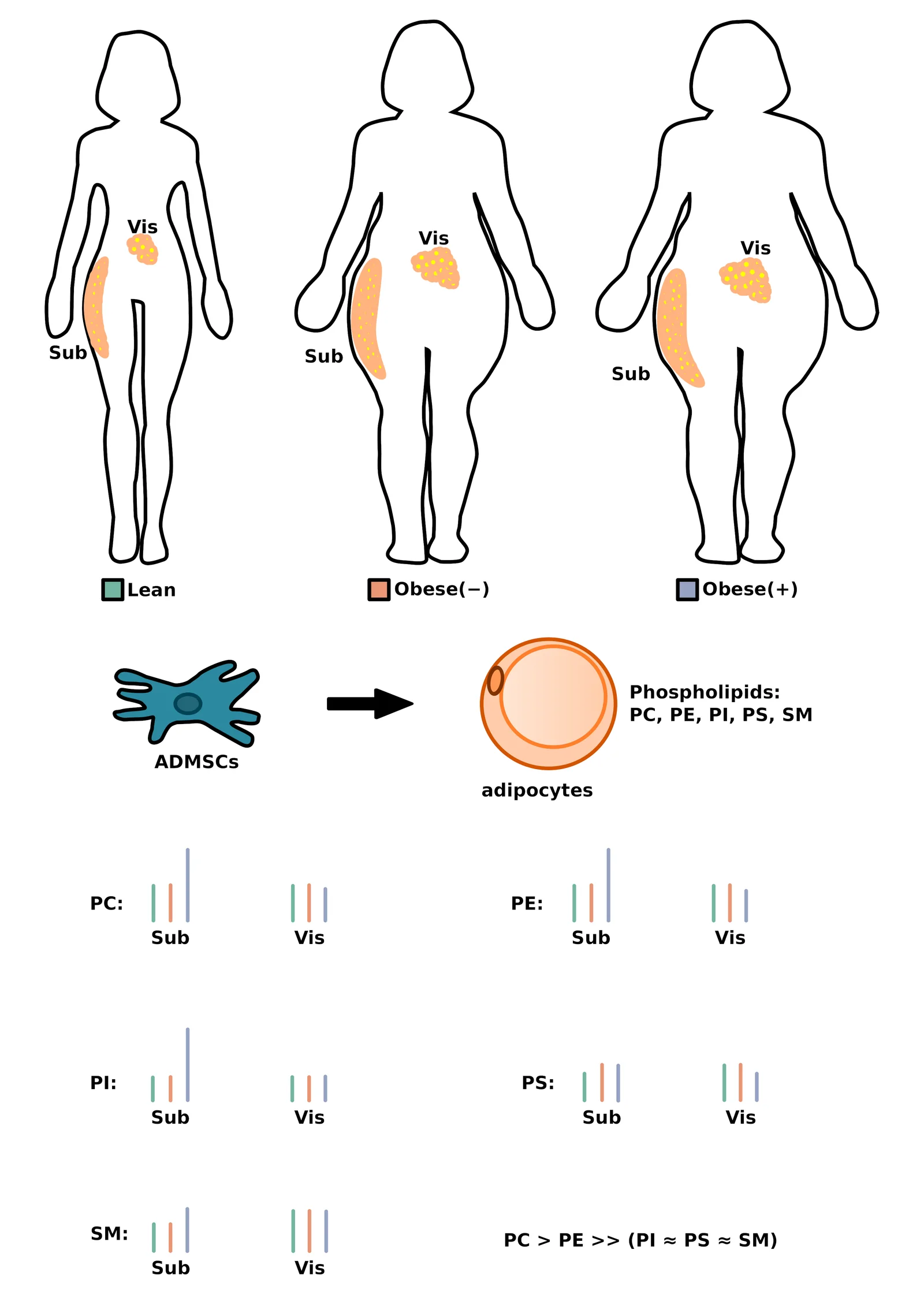
Graphical abstract of the most important obtained results. ADMSCs: adipose-derived mesenchymal stem cells; Obese(−): adipocytes differentiated from ADMSCs of patients with obesity but without metabolic syndrome; Obese(+): adipocytes differentiated from ADMSCs of patients with obesity with accompanying metabolic syndrome; Sub: subcutaneous adipose tissue; Vis: visceral adipose tissue; PC: phosphatidylcholine; PE: phosphatidylethanolamine; PI: phosphatidylinositol; PS: phosphatidylserine; SM: sphingomyelin.

## Data Availability

The data presented in this study are available upon request from the corresponding author.

## Supplementary Materials

The following supporting information can be downloaded at: https://www.mdpi.com/article/10.3390/cells15191725/s1. Figure S1: Exemplary TLC (thin layer chromatography) plate; Table S1: Phospholipids concentrations—total contents and fatty acids composition.

## Abbreviations

ADMSC	adipose-derived mesenchymal stem cells
ANOVA	analysis of variance
ATP	adenosine triphosphate
BMI	body mass index
CER	ceramides
DAG	diacylglycerols
FA	fatty acids
GLUT-4	glucose-sensitive transporter 4
IR	insulin resistance
LPCAT3	lysophospholipid acyltransferase
MADM	mesenchymal stem cell adipogenic differentiation medium
MSCM	mesenchymal stem cell medium
NEFA	non-esterified fatty acids
PC	phosphatidylcholine
PE	phosphatidylethanolamine
PI	phosphatidylinositol
PS	phosphatidylserine
SAT	subcutaneous adipose tissue
SM	sphingomyelin
sWAT	subcutaneous white adipose tissue
TAG	triacylglycerols
TLC	thin-layer chromatography
VAT	visceral adipose tissue
WAT	white adipose tissue
WHO	World Health Organization
