# Supplementary material for: Significant Alterations in Phospholipid Content and Composition Contributing to Metabolic Dysfunction in ADMSC-Derived Adipocytes
Source: Cells. 2026 Sep 22;15(19):1725. doi: 10.3390/cells15191725 (PMC13664016; doi:10.3390/cells15191725)
Supplement: Supplementary file 1 [file cells-15-01725-s001.zip › supplementary_Figure S1.pdf]

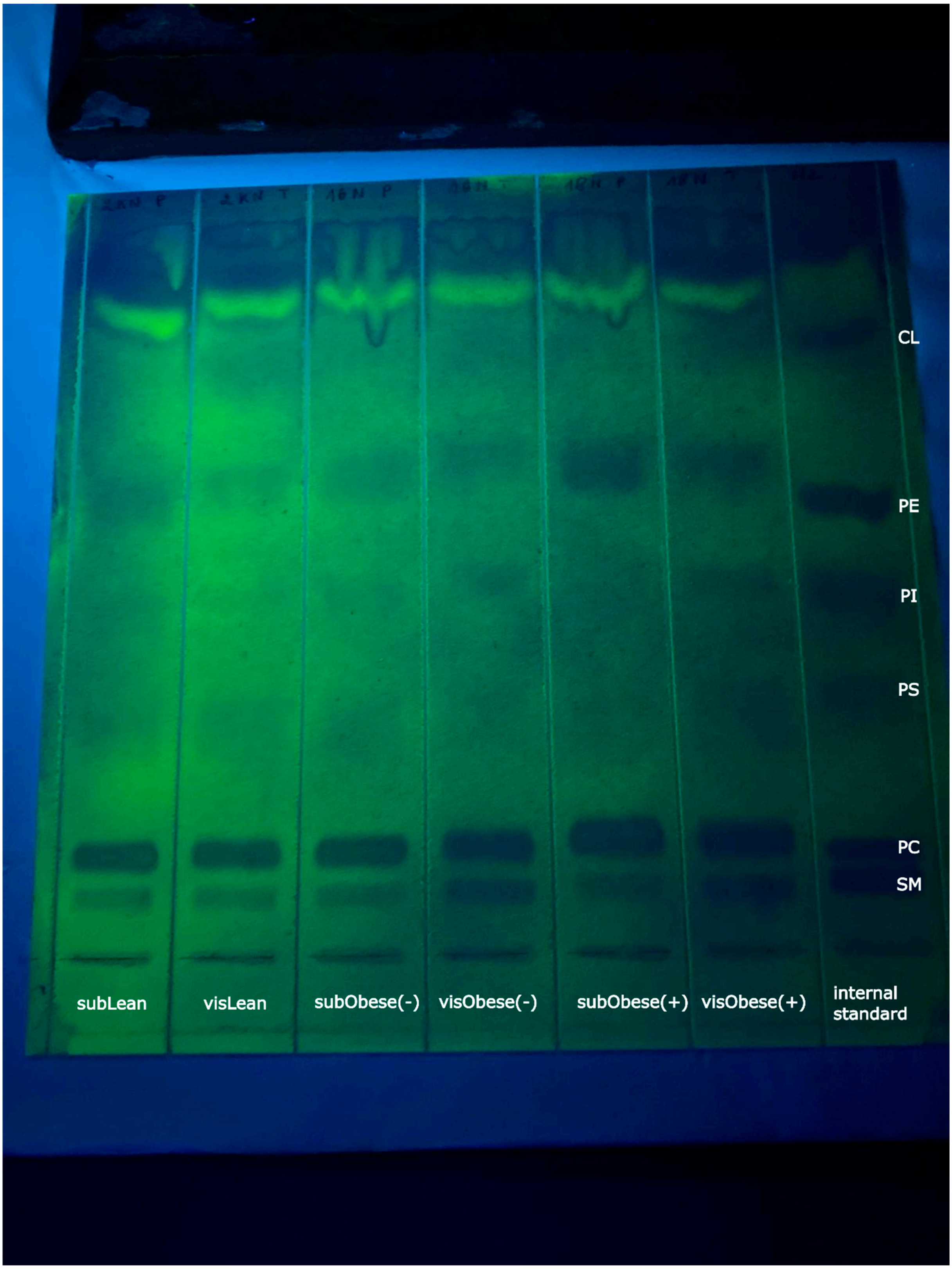

Supplementary Figure S1. Exemplary TLC (thin layer chromatography) plate. Lipid bands were made visible by spraying plates with a 0.2% solution of 3'7'-dichlorofluorescin in methanol and recognized

under ultraviolet light using standards on the plates. Obese (−): adipocytes differentiated from ADMSCs of patients with obesity but without metabolic syndrome, Obese (+): adipocytes differentiated from ADMSCs of patients with obesity with accompanying metabolic syndrome, Sub – subcutaneous adipose tissue, Vis – visceral adipose tissue, PC – phosphatidylcholine, PE – phosphatidylethanolamine, PI – phosphatidylinositol, PS – phosphatidylserine, SM – sphingomyelin, CL - cardiolipin
